# On the Feasibility of Detecting Faults and Irregularities in On-Load Tap Changers (OLTCs) by Vibroacoustic Signal Analysis

**DOI:** 10.3390/s24247960

**Published:** 2024-12-13

**Authors:** Hassan Ezzaidi, Issouf Fofana, Patrick Picher, Michel Gauvin

**Affiliations:** 1 Canada Research Chair Tier 1 in Aging of Oil-Filled Equipment on High Voltage Lines (ViAHT), University of Quebec at Chicoutimi, Chicoutimi, QC G7H 2B1, Canada; 2 Hydro Québec’s Research Institute, Varennes, QC J3X 1S1, Canada; picher.patrick@hydroquebec.com (P.P.); gauvin.michel@hydroquebec.com (M.G.)

**Keywords:** power transformer, OLTC, vibroacoustic signal, measure-based band tolerance, amplitude modulation, Gaussian carrier, envelope, statistics

## Abstract

Unlike traditional tap changers, which require transformers to be de-energized before making changes, On-Load Tap Changers (OLTCs) can adjust taps while the transformer is in service, ensuring continuous power supply during voltage regulation. OLTCs enhance grid reliability and support load balancing, reducing strain on the network and optimizing power quality. Their importance has grown as the demand for stable voltage and the integration of renewables has increased, making them vital for modern and resilient power systems. While enhanced OLTCs often incorporate stronger materials and improved designs, mechanical components like contacts and diverter switches can still experience wear over time. This can result in longer maintenance intervals. In the era of digitalization, advanced diagnostic techniques capable of detecting early signs of wear or malfunction are essential to enable preventive maintenance for these important components. This contribution introduces a novel method for detecting faults and irregularities in OLTCs, leveraging vibroacoustic signals to enhance OLTC diagnostics. This paper proposes a tolerance-based approach using the envelope of vibroacoustic signals to identify faults. A significant challenge in this field is the limited availability of faulty signal data, which hinders the performance of machine learning algorithms. To address this, this study introduces a nonlinear model utilizing amplitude modulation with a Gaussian carrier to simulate faults by introducing controlled distortions. The dataset used in this study, with data recorded under real operating conditions from 2016 to 2023, is free of anomalies, providing a robust foundation for the analysis. The results demonstrate a marked improvement in the robustness of detecting simulated faults, offering a promising solution for enhancing OLTC diagnostics and preventive maintenance in modern power systems.

## 1. Introduction

On-Load Tap Changers (OLTCs) are crucial for regulating the voltage ratio of an electrical transformer while it is operating. This is achieved through the operation of a selector switch. During the process of changing taps, both fixed contacts and mobile components generate a vibroacoustic signal. Variations in the frequency and amplitude of this signal are believed to contain specific information about the electrical and mechanical characteristics of the equipment. Any anomaly in the OLTC results in changes to the shape of the vibroacoustic signal, highlighting deviations from normal operation.

Vibroacoustic signals, recorded by an accelerometer, are often processed by extracting their envelopes. This reduces data volume while capturing a signature signal. The positions and timing of peaks in the envelope signal correspond to mechanical impacts within the OLTC’s fixed and mobile contact systems. Envelope peaks may overlap when contacts are close or synchronize their movements.

Several research projects have concentrated on developing automated methods for detecting or predicting faults in OLTC systems (e.g., [1,2,3,4,5]). Vibroacoustic measurement has proven particularly useful for detecting mechanical issues or wear and tear, which traditionally required expert visual inspections that were often subjective and dependent on the assessor’s experience. To maximize the potential of this technique for field diagnostics, it is crucial to develop sophisticated algorithms that can process and analyze data effectively, enabling more accurate and automated assessments [6]. However, acquiring suitable data for this research direction poses challenges. It is relatively straightforward to collect data from OLTCs operating under normal conditions over several years. In contrast, obtaining data with anomalies is complex [7].

In industrial sectors, when anomalies occur, components in the switching system are often replaced with worn equivalents to capture the vibroacoustic signals they generate.

As a result, data on faulty vibration signals remain limited and statistically non-representative. This scarcity of anomaly data complicates the development and testing of automated fault detection methods for OLTC systems. To address the scarcity of fault-state vibration signal data, some research efforts have turned to physical modeling approaches for OLTCs. These methods typically employ finite element and dynamic models to simulate various anomalies. By utilizing these models, researchers can generate large datasets of vibration signals under faulty conditions [8]. This approach could help overcome the challenge of limited real-world anomaly data and facilitate the development and testing of automated fault detection methods for OLTCs. It is important to note that physical modeling is inherently complex and challenging. Hence, a digital twin approach based on data-driven dynamic model updating was proposed to estimate the uncertain operating conditions and the mechanical state transition [9]. The model learns from the vibroacoustic signal without requiring prior knowledge of fault states. Recently, a linear model based on the timing shifts in the diverter’s operating sequence was proposed as an indicator for detecting contact wear [6]. All experimental data used to study contact wear levels were collected in the laboratory and employed to train the proposed model.

The vibroacoustic signal measurement of OLTCs is a non-invasive technique designed to capture various signatures that characterize mechanical events (such as contact and movement) and electrical events, which occur at lower and higher frequency bands, respectively. Extracting characteristic attributes and signatures from envelope or vibration signals has been explored using various techniques such as empirical mode decomposition, variational decomposition, wavelet decomposition, and the analysis of temporal and frequency parameters [10,11,12].

The Short-Time Fourier Transform (STFT) and Synchrosqueezed Wavelet Transform (SWT) were applied to vibroacoustic signals to capture both global and local features. These features were then analyzed using a convolutional neural network (CNN) with a support vector machine (SVM) as the output layer. Experimental data were collected under laboratory conditions, including normal operation, upper and lower static contact looseness, insulated panel looseness, moving contact looseness, contact erosion/wear, and jamming [4]. To enhance the feature extraction process, an improved empirical mode decomposition (EMD) energy spectrum algorithm was employed [5], along with an ensemble empirical mode decomposition (EEMD) combined with wavelet thresholding to denoise OLTC acoustic signals. This approach not only improved signal decomposition but also facilitated a better diagnosis of mechanical issues. Similarly, to further improve the detection of mechanical faults in OLTCs, a variational mode decomposition (VMD) approach combined with a weight divergence method was proposed, optimized using the Harmony Search (HS) algorithm to fine-tune parameters [13].

The location and tracking of the main peaks in the signal envelopes were analyzed to detect faults for condition monitoring. According to Rivas et al. [3], the time lag between bursts and their absence was identified as the most reliable indicator for detecting tap selector anomalies. In this experiment, various failures in the tap selector were physically simulated, including loose contact springs, broken contact bars, worn tap selector contacts, and arcing.

Machine learning (ML) has rapidly advanced in recent years, offering significant improvements in industrial applications, particularly in fault diagnosis and detection. Several types of supervised and unsupervised learning algorithms have been proposed in machine learning for classifying anomalies. For example, the following are proposed:

- In aero-engine bearings with limited samples, innovative techniques like transfer learning data augmentation and few-shot learning are addressing data scarcity issues [14]. These methods can achieve high-accuracy fault diagnosis even with limited samples by leveraging pre-trained models from similar tasks or artificially expanding the available data. Additionally, combining deep learning with signal processing methods (like wavelet transforms or time–frequency analysis) further enhances the accuracy and robustness of fault detection in bearing systems.
- Meanwhile, in wind turbines, improved methods such as multiscale fuzzy entropy (MFE) combined with ML are providing more reliable and early fault detection [15]. Improved MFE can capture subtle changes in the turbine’s condition, such as early signs of mechanical wear or electrical faults, which may not be detectable through conventional methods. Machine learning models, such as support vector machines (SVMs) and neural networks, can be integrated with MFE to automatically classify fault types and provide actionable insights for predictive maintenance. This combination allows for a more accurate and early detection of faults, even in complex, nonlinear systems, thus improving the operational efficiency of wind turbines.

These advancements are paving the way for smarter, more efficient industrial systems that can reduce maintenance costs and improve system reliability.

These include neural networks, support vector machines, k-means clustering, and statistical and temporal threshold methods [16,17,18]. These approaches aim to effectively detect and classify anomalies in OLTC systems based on the extracted signal features and patterns. Recently, a model combining an autoencoder with a kernel density estimation approach was applied to continuously measured vibration signal envelopes. The model was trained on a normal dataset, and the reconstruction error was used to compute anomaly scores, with a static threshold for fault detection [16].

This study aims to improve preventive maintenance strategies, reduce system downtime, and optimize the operation of OLTCs by providing a more effective, non-invasive diagnostic tool. This study’s novelty lies in its innovative approach to OLTC diagnostics by leveraging simulated faults through the modulation of clean vibroacoustic signal envelopes using Gaussian functions. The dataset, covering 8 years of operation starting in 2016, includes 22,000 tap operations and 35,000 switching operations, providing a comprehensive basis for this study. This method addresses the challenge of insufficient faulty data for training machine learning models, a common limitation in predictive maintenance and fault detection systems. By introducing controlled distortions into the clean signals, this study simulates fault conditions, creating a rich dataset for anomaly detection without relying on prior knowledge of fault states, parameter vectors, or complex system models.

## 2. Methodology

### 2.1. Database and Processing

**Figure 1:**
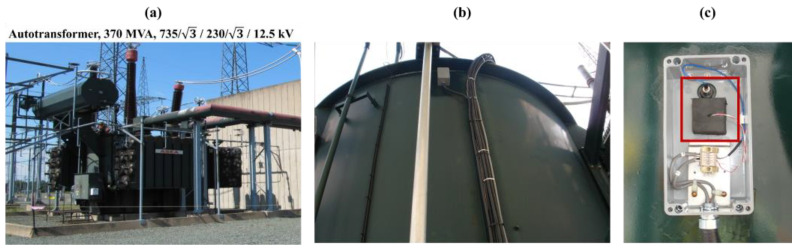
The installation of the accelerometer and temperature and current clamp sensors: (**a**) an overview of the autotransformer, (**b**) the installation of the sensor on the transformer tank, and (**c**) the box where the accelerometer and temperature sensors are installed is highlighted with a red rectangle.

**Figure 2:**
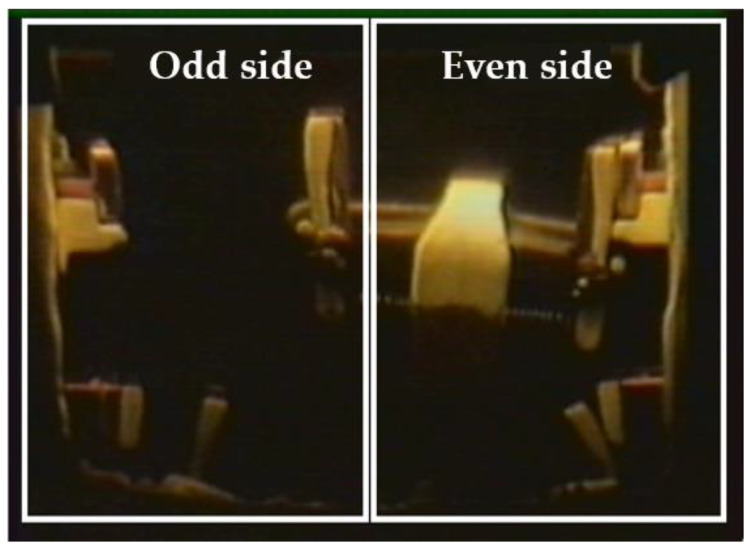
Internal mechanical design of OLTC.

An OLTC monitoring system developed at the Hydro Quebec’s Research Institute (IREQ) was installed on nine single-phase autotransformers rated at 370 MVA with voltage ratings of 735/√3 kV on the primary side and 230/√3 kV on the secondary side, currently in operation on Hydro-Québec’s electric network (Figure 1). Vibration signal envelopes are continuously measured and recorded using an accelerometer. A detailed description of this monitoring system can be found in [7,19]. The ABB-UC OLTC model [7,19,20] is a type of On-Load Tap Changer (OLTC) integrated within the transformer tank. In this model, two selectors—designated as odd and even—manage connections, with a switch enabling transitions between these sides (Figure 2).

It should be noted that the measured vibration signals are complex and contain redundant information. To address this, a signal envelope extraction technique is employed to isolate the envelope from each vibration signal. In this study, envelope extraction is achieved using a combination of the Hilbert transformer and a Low-Pass Filter. Subsequently, first-order time realignment and moving average techniques are applied to mitigate the effects of temperature and reduce natural variations between the acoustic signatures of a tap changer, respectively [7].

This research focuses on studying vibroacoustic signal envelopes, specifically during the switching operations of three OLTCs corresponding to three sister units continuously monitored from 2016 to 2023. These transformers are designated as T3A, T3B, and T3C throughout this paper, and they utilize ABB UC OLTCs. Based on the mechanical design of their main switching contacts (odd and even positions), each dataset can be categorized into these two groups. Consequently, the analysis involves six datasets: T3A-odd, T3A-even, T3B-odd, T3B-even, T3C-odd, and T3C-even.

### 2.2. Proposed Model Distortion

Real fault data represent a significant gap in training and testing the various approaches proposed in the scientific literature for the diagnosis and predictive maintenance of OLTCs [9]. In many studies, faulty signals used for testing were typically generated through laboratory experiments, where defective components were deliberately introduced. While useful, this method is limited by the relatively small number of available data. Alternatively, simulations based on models have also been employed, but they may lack the complexity and variety of real-world fault scenarios.

Since the signal envelope is utilized in this work, some mechanical distortions are assumed to be introduced by shifting and/or attenuating the peaks of the main lobes of the envelope, eliminating the need to model the physical behavior of its components. Therefore, amplitude modulation is applied to introduce distortions to the recorded envelope of the vibroacoustic signal. This is achieved by computing the product with a carrier signal defined by a Gaussian function, as follows:

(1) $$g\left(x\right)=\frac{1}{c}exp(-\frac{{\left(x-\mu \right)}^{2}}{{2\sigma }^{2}})$$

where x is a vector of N samples linearly spaced over the interval [9], the variable μ represents the mean vector, σ represents the standard deviation vector, and c is used as a normalization factor. Different combinations of the mean (μ) and standard deviation (σ) values were selected to generate a set of 49 Gaussian functions, each with distinct shapes and centers: μ = {−2, –1, −0.5, 0, 0.5, 1, 2} and σ = {0.1, 0.5, 1, 1.5, 1.7, 2, 2.2}.

**Figure 3:**
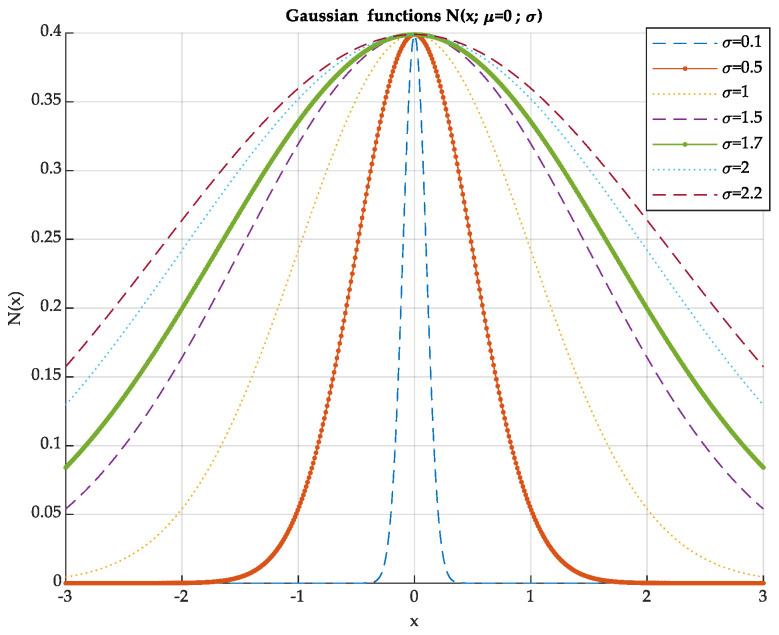
Gaussian functions.

Figure 3 illustrates various shapes of centered Gaussians with different variances. The horizontal axis corresponds to the number of envelope samples, which is fixed at N = 500 for all data presented in this study.

For instance, when μ = 0, the distortions introduced are symmetrical and increase with the variance σ. When the mean μ is shifted to the left or right of the center, the distortions in the signal envelope become asymmetrical. A small variance (σ) results in a selective Gaussian shape, leading to highly nonlinear distortions, particularly at the beginning and end of the signal envelope sequence. Conversely, a large variance makes the Gaussian shape wider and less selective, resulting in smoother distortions.

The matching problem, which can be seen as a scale distortion (stretching or compression) between the reference envelope signal and the test envelope signal, is intended to be minimized by applying the dynamic time alignment algorithm. However, when the distortions in the positions of the main peaks between the envelopes are significant, the similarity distance measurement can easily identify dissimilarities. This scenario is modeled by modulating the original signal with narrow Gaussian functions. On the other hand, modulation with wide Gaussian functions results in small attenuations (distortions), which become more challenging to detect.

**Figure 4:**
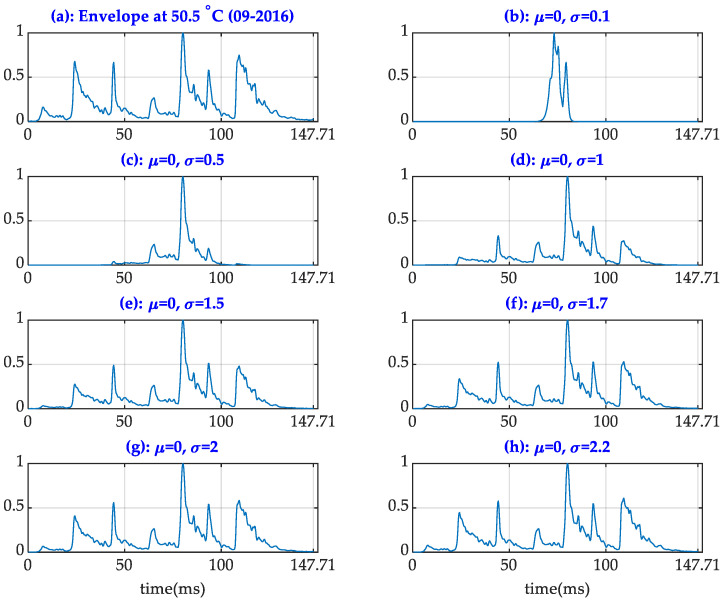
Original and distorted envelopes.

Figure 4 illustrates examples of an original (clean) signal envelope and its corresponding versions with distortions. The distortions in the envelope signals are pronounced when a narrow Gaussian function is applied, whereas distortions are minimal when using wider Gaussian shapes (σ ≥1.5). To streamline this study, the investigated mean and variance values were limited to the following sets: μ = {−1, −0.5, 0, 0.5, 1} and σ = {1.7, 2, 2.2}.

### 2.3. Prototype of Envelope Signal

In practice, diagnosing the operational state of OLTCs involves analyzing the shape of envelope signals over time. A signal envelope is extracted and compared to a prototype envelope recorded during a known good operating condition to achieve this.

In this study, the prototype signal is computed as the average envelope over the first 12 months of operation. This timeframe accounts for variations and seasonal temperature impacts on the vibroacoustic signals. Therefore, a single prototype envelope represents each type of OLTC (even and odd positions) within a family.

To detect and diagnose faults or irregularities in equipment, each envelope signal is aligned with its corresponding prototype using the cross-correlation method. The similarity between the aligned pairs of envelopes is quantified by calculating the Euclidean distance. Fault detection is accomplished by evaluating these Euclidean distance measurements using the anomaly indicators outlined in the subsequent section.

### 2.4. Indicator Measures of Anomalies

In stock transactions, indicators are quantitative measures used to understand and monitor price trends and manage market risks effectively. These indicators encompass various types, such as oscillators (e.g., stochastic oscillators), trend indicators (e.g., moving average), volatility indicators (e.g., Bollinger bands), volume indicators, and momentum indicators. Inspired by the parabolic SAR indicator [21] commonly employed by traders to ascertain trend direction, a series of indicators is proposed and evaluated in this study to detect potential anomalies in OLTCs using all available data. These indicators are defined by the following equations:

(2) $$I_{mean}\left(i\right)={\vec{I}}_{mean}+\beta \left(\left[x\left(i\right)-{\vec{I}}_{mean}\right]\right)$$

(3) $$I_{min}\left(i\right)=I_{min}\left(i-1\right)+\beta (x\left(i\right)-I_{min}\left(i-1\right))$$

(4) $$I_{max}\left(i\right)=I_{max}\left(i-1\right)+\beta \left(x\left(i\right)-I_{max}\left(i-1\right)\right)$$

(5) $$I_{mavg}\left(i\right)=\frac{\sum_{k=i}^{i-N}x\left[k\right]}{N}$$

where

$x\left[i\right]$ is the current time of the Euclidian distance measured between the reference and the test envelope.
$I_{mean}\left[i\right]$ denotes the current time of the indicator measure (idem for $I_{max}\left[i\right],I_{min}\left[i\right]\;and\;I_{mavg}\left[i\right])$.
β represents the forgetting factor fixed at 0.01.
${\vec{I}}_{mean}$ indicates a vector of the last 14 measurements of the indicator $I_{mean}\left[i\right]$.
Max(), Min(), and Mean() are, respectively, the maximum, minimum, and mean operators.
$I_{mavg}\left(i\right)$ denotes the mean calculated over a sliding window of the current sample k and N previous samples where N is evaluated over different sizes {7,14,30}.

In the stock market, the β factor is often referred to as an acceleration factor, with its values being dynamically adjusted. In our case, however, the forgetting factor β is fixed, and the recursive expression of the proposed indicator takes the form of a geometric sequence with a ratio of β, as follows:

(6) $$I\left(n\right)=\beta \sum_{n=0}^{n=N}x\left(n\right){\left(1-\beta \right)}^{N-n}$$

To prevent the saturation of the indicator, the contribution of distant samples at time i−Ni-N can be subtracted from the most recent indicator measurements. This approach helps limit the prediction within the sliding window, ensuring that older data have a diminishing effect on the current calculation.

## 3. Results and Discussion

### 3.1. Graphical Analysis

The proposed indicators were rigorously tested using the data outlined in Section 2.1. Original data were recorded under real operating conditions for three OLTC families. To expand the dataset, each family’s data underwent the introduction of nonlinear distortions, resulting in the creation of 15 additional data groups, as detailed in Section 2.2.

**Figure 5:**
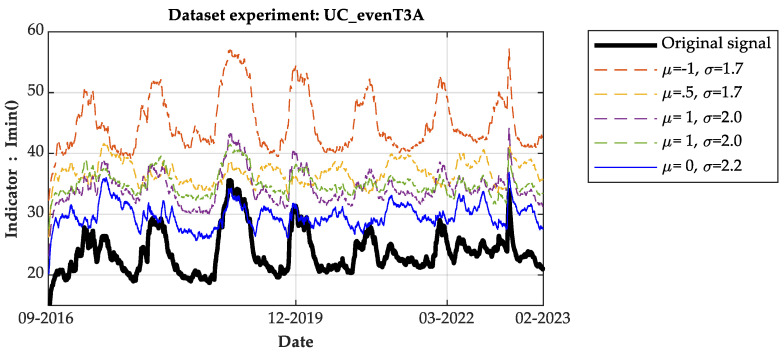
Indicator measure I_min () using dataset of T3A family.

Figure 5 presents the measurements of the Imin() indicator for family A (even type) applied to the original signal under normal operating conditions, along with five corresponding versions that include various distortion modulations.

**Figure 6:**
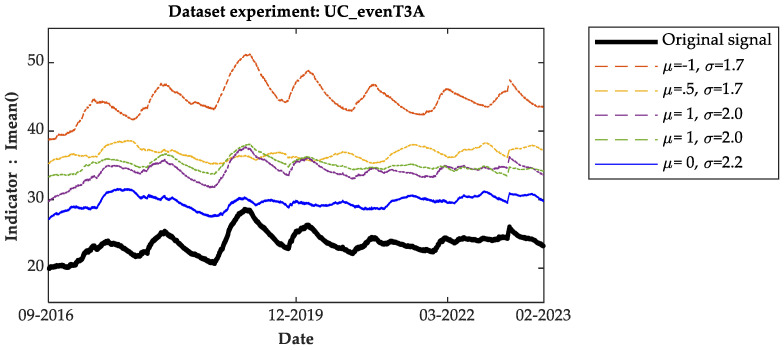
Indicator measure I_mean () using dataset of T3A family.

**Figure 7:**
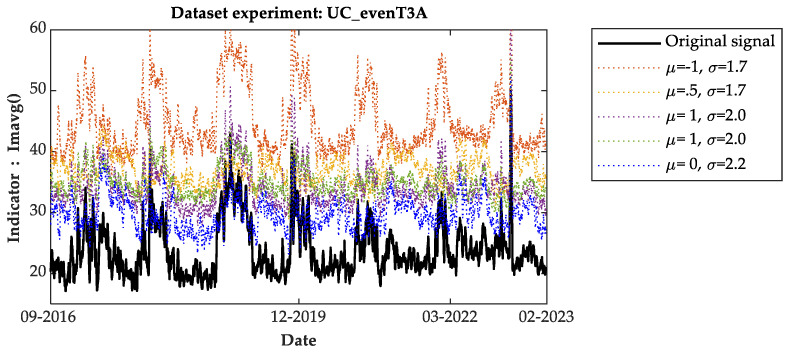
Indicator measure mobile average using dataset of T3A family.

Figure 6 and Figure 7 present the measurements of the Imean() indicator and moving average, respectively. The bold blue lines in Figure 5 and Figure 6 represent the measurements of the original data without any distortions from 2016 to 2023.

This visual representation illustrates the effectiveness of the proposed indicators in analyzing the data and detecting anomalies in OLTCs, both under real and simulated conditions. To streamline the graphs’ content, only five distortion options are presented.

From the analysis of the indicators over time, it is evident that each indicator shows a consistent trend with minimal fluctuations and periodic variations likely influenced by temperature effects. The amplitude levels generally increase across all indicators, with the lowest amplitude observed in the reference signal. Graphically, the moving average measurements reveal frequent shifts in the lowest amplitude levels between the reference and distorted signals. Despite these shifts, the amplitude levels consistently remain stable and lower for the reference signal throughout the observation period, particularly noticeable in the Imean() indicator. This observation suggests that the reference signal consistently maintains a lower amplitude compared to the distorted signals across various indicators, highlighting its stability and reliability as a benchmark in assessing OLTC anomalies.

As per the fault detection criterion defined here, any measurement falling within the tolerance band, defined by the maximum and minimum values of the reference signal indicator, signifies the normal operation of the OLTC. Conversely, measurements outside this band indicate the presence of an anomaly in the OLTC, necessitating predictive maintenance. It is worth noting that the measurements from the I_min and I_max indicators are comparable. Therefore, for the sake of simplicity and clarity in this work, the results using the I_max indicator are not presented.

This approach ensures a straightforward method for identifying deviations from normal operating conditions in OLTCs, facilitating timely maintenance interventions to prevent potential failures.

Similarly, tolerance band thresholds based on the proposed indicators were separately determined for each dataset within families A, B, and C, including both the even and odd types. To evaluate and compare performance, a score error was computed for all generated envelope signals with distortions.

The score error is defined as the ratio of the number of envelope signals classified as normal (no anomalies detected) while actually originating from an envelope signal with anomalies divided by the total number of envelope signals used in the test. This score error ratio provides a normalization factor to assess the accuracy of anomaly detection across different datasets and types of OLTCs.

### 3.2. Compilation of Results 

Table 1 illustrates the scores obtained using the I_min() indicator on the data recorded from September 2016 to December 2023. The first column displays the parameter values (μ, σ) of the Gaussian function used to generate envelope signals with distortions, specifically highlighting parameters where anomalies were detected. The second, third, and fourth columns present the score errors obtained for families A, B, and C, respectively.

This table provides a detailed comparison of anomaly detection performance across different parameter settings, emphasizing the effectiveness of the I_min() indicator in identifying deviations from normal operation in OLTCs within each family.

The results indicate that anomalies are primarily detected when the variance in Gaussian functions is high. This observation can be attributed to the characteristics of Gaussian functions: When the variance is high, the Gaussian curve is wider and flatter, resulting in less pronounced distortions in the envelope signals. Conversely, when the variance is low, the Gaussian curve is narrower and taller, leading to more significant distortions.

Interestingly, shifting the center (mean) of the Gaussian function to different positions does not appear to affect the performance scores of anomaly detection significantly. This suggests that the asymmetrical distortions caused by varying the mean do not substantially impact the indicator’s ability to detect anomalies compared to the variance parameter.

In summary, the results underscore the sensitivity of anomaly detection to the variance parameter in Gaussian function modulation, highlighting the importance of this parameter in simulating and detecting anomalies in OLTCs using the I_min() indicator.

Table 2 illustrates the score errors obtained with the I_mean() indicator using the same dataset. This table shows minimal errors in anomaly detection scores. Specifically, significant confusion in detecting anomalies is observed with distortions introduced by the Gaussian function centered at 2.2. The error scores are 15.4% and 19.7% for families A and C, respectively, and 0% for family B, encompassing both the even and odd types.

This table underscores the challenges encountered with anomaly detection using the I_mean() indicator, particularly when dealing with distortions generated by Gaussian functions centered at higher values like 2.2. The varying performance across different families and types indicates the indicator’s sensitivity to specific parameter settings, affecting its ability to accurately detect anomalies in OLTCs. It would be interesting to validate the I_mean() indicator with actual defect data and assess the context of its use. The temperature factor on the shape has been demonstrated in several studies, and it would be valuable to explore its use as a weighting factor to minimize fluctuations in similarity measurements.

## 4. Conclusions

In this study, several indicators were introduced to quantify the Euclidean distance measure of vibroacoustic signal envelopes to enhance the diagnostic process for On-Load Tap Changers (OLTCs). The dataset used for experimentation was based on real operational conditions (including 22,000 tap operations and 35,000 switching operations) collected over 8 years, providing a rich and comprehensive foundation for analysis. To further enrich the dataset, a method was applied that involved modulating signals with Gaussian functions of varying means and variances, effectively simulating anomalies in OLTCs.

Among the proposed indicators, one based on the mean operator demonstrated particularly strong performance. This technique effectively simulates a wide range of anomalies that can occur in OLTCs, allowing for a more realistic representation of operational faults. This approach demonstrated exceptional robustness in handling distortions and was highly efficient in detecting anomalies using the simulated data. Given these promising results, it is expected that this method will be equally effective in identifying anomalies in real-world scenarios, where the complexity of and variability in faults are more pronounced. This makes the proposed approach a valuable tool for the ongoing development of diagnostic and predictive maintenance strategies for OLTCs.

While simulated data are valuable for developing models, they cannot fully replicate the variability in and unpredictability of real-world environments, which is why the careful validation and continual refinement of the model are necessary to improve its accuracy. Additionally, incorporating long-term operational data is critical to enhancing reliability predictions.

From a practical perspective, the findings highlight the critical role of the tolerance scheme developed in this study. This tolerance scheme, based on vibroacoustic signal analysis, serves as a valuable tool for engineers in the energy sector. By offering a more reliable method for anomaly detection and diagnostics, it can support better decision-making, reduce unplanned downtime, and extend the operational lifespan of OLTCs. Engineers in the field are encouraged to integrate this approach into their condition monitoring systems, as it provides a non-invasive, cost-effective solution for improving OLTC performance and overall grid reliability.

## Figures and Tables

**Table 1:** The scores obtained using the I_min() indicator on the data recorded from September 2016 to December 2023.

(μ, σ)	Score Error of Indicator Imin().					
	T3A		T3B		T3C	
	Even	Odd	Even	Odd	Even	Odd
(−1, 1.7)	0%	0%	0%	0%	0%	0%
(−0.5, 1.7)	0%	3%	0%	0%	5%	3%
(0, 1.7)	33%	16%	0%	9%	26%	8%
(0.5,1.7)	2%	0%	0%	1%	9%	0%
(1, 1.7)	0%	0%	0%	0%	0%	0%
(−1, 2)	0%	10%	0%	0%	42%	25%
(−0.5, 2)	66%	66%	27%	75%	84%	76%
(0, 2)	85%	79%	44%	93%	88%	72%
(0.5, 2)	75%	36%	30%	32%	78%	4%
(1, 2)	4%	0%	0%	0%	5%	0%
(−1, 2.2)	20%	37%	15%	28%	68%	55%
(−0.5, 2.2)	92%	81%	85%	90%	98%	87%
(0, 2.2)	99%	86%	91%	98%	100%	85%
(0.5, 2.2)	98%	67%	71%	72%	99%	34%
(1, 2.2)	58%	10%	17%	13%	69%	0%

**Table 2:** The score errors obtained with the I_mean() indicator using the same dataset.

(μ, σ)	Score Error of Indicator Imin().					
	T3A		T3B		T3C	
	Even	Odd	Even	Odd	Even	Odd
(−1, 1.7)	0%	0%	0%	0%	0%	0%
(−0.5, 1.7)	0%	0%	0%	0%	0%	0%
(0, 1.7)	0%	0%	0%	0%	0%	0%
(0.5,1.7)	0%	0%	0%	0%	0%	0%
(1, 1.7)	0%	0%	0%	0%	0%	0%
(−1, 2)	0%	0%	0%	0%	0%	0%
(−0.5, 2)	0%	0%	0%	3%	2%	0%
(0, 2)	0%	8%	0%	4%	0%	0%
(0.5, 2)	0%	0%	0%	0%	0%	0%
(1, 2)	0%	0%	0%	0%	0%	0%
(−1, 2.2)	0%	4%	0%	0%	2%	0%
(−0.5, 2.2)	3%	21%	0%	47%	6%	3%
(0, 2.2)	13%	41%	0%	52%	16%	0%
(0.5, 2.2)	4%	0%	0%	4%	10%	0%
(1, 2.2)	0%	0%	0%	0%	0%	0%

## Data Availability

The datasets presented in this article are not readily available because the data are part of an ongoing study.
